# "Tied together like a woven hat:" Protective pathways to Alaska native sobriety

**DOI:** 10.1186/1477-7517-1-10

**Published:** 2004-11-17

**Authors:** Gerald V Mohatt, S Michelle Rasmus, Lisa Thomas, James Allen, Kelly Hazel, Chase Hensel

**Affiliations:** 1 University of Alaska, Box 757000, Fairbanks, Alaska, 99775-700, USA; 2 University of Washington, Box 351525, Seattle WA 98195, USA; 3 Metropolitan State University, 730 Hennepin Ave., Minneapolis, MN 55403-1897, USA; 4 Psychosocial Center for Refugees, University of Oslo, Boks 1072 Blinden, NO 0316, Oslo, Norway

## Abstract

**Background:**

The People Awakening Project (1RO1 AA 11446-03) had two purposes, completed in Phase I and Phase II of the project. The purpose of Phase I was to complete a qualitative study; the research objective was discovery oriented with the specific aim of identification of protective and recovery factors in Alaska Native sobriety. Results were used to develop a heuristic model of protective and recovery factors, and measures based on these factors. The research objective of Phase II was to pilot these measures and provide initial validity data.

**Methods:**

Phase I utilized a life history methodology. People Awakening interviewed a convenience sample of 101 Alaska Natives who had either recovered from alcoholism (n = 58) or never had a drinking problem (n = 43). This later group included both lifetime abstainers (LAs) and non-problem drinkers (NPs). Life histories were transcribed and analyzed using grounded theory and consensual data analytic procedures within a participatory action research framework. Analyses were utilized to generate heuristic models of protection and recovery from alcohol abuse among Alaska Natives.

**Results:**

Analyses generated a heuristic model of protective factors from alcohol abuse. The resulting multilevel and multi-factorial model describes interactive and reciprocal influences of (a) individual, family, and community characteristics; (b) trauma and the individual and contextual response to trauma, (c) experimental substance use and the person's social environment; and (d) reflective processes associated with a turning point, or a life decision regarding sobriety. The importance of cultural factors mediating all these protective processes is emphasized. For NPs, the resilience process drew from personal stores of self-confidence, self-efficacy, and self-mastery that derived from ability to successfully maneuver within stressful or potentially traumatizing environments. In contrast, for many LAs, efficacy was instead described in more socially embedded terms better understood as communal mastery. One style of mastery is more associated with individualistic orientations, the other with more collectivistic. Future research is needed regarding the generalizeability of this group difference.

**Conclusions:**

Results suggest that preventative interventions should focus on intervening simultaneously at the community, family, and individual levels to build resilience and protective factors at each level. Of particular importance is the building of reflexivity along with other cognitive processes that allow the individual to think through problems and to reach a life decision to not abuse alcohol.

## Background

Many American Indian and Alaska Native people experience problems with alcohol abuse that lead to social, psychological, and physical problems [1-3]. Unfortunately, little is known about American Indian or Alaska Native people who live sober and healthy lives. This paper presents initial findings from the People Awakening Project (PA), a collaborative study involving the Alaska Native community, and Native and non-Native university researchers. The goal of PA was to provide an Alaska Native understanding of the sobriety process. In earlier work, we provided a detailed description of PA's focus on cultural and spiritual understandings of sobriety [4], and its use of participatory research methodologies with Alaska Natives [5]. Sobriety in the addiction literature is generally defined as total abstinence following a period of alcohol abuse and/or dependence. However, many Alaska Natives also consider life-long abstinence, as well as non-abusive or moderate use of alcohol, as examples of a sober lifestyle. PA has adopted this broader definition of sobriety.

Recent research on resilience identifies and describes protective factors that moderate risk and adverse environmental circumstances; this work has relevance to understanding the sobriety process [6-10]. Resilience is "a capacity that develops over time in the context of person-environment interactions" [11] (p. 517). Protective factors are those attributes that contribute to this capacity, and include those "individual characteristics or environmental conditions that help children and youth resist or otherwise counteract the stress to which they were exposed. They delay, suppress, or neutralize negative outcomes" [12] (p. 4). Protective factors can be grouped according to three broadly conceived categories [13-15]: (a) internal or dispositional attributes of the individual, such as sociability, intelligence, social competence, and internal locus of control; (b) familial attributes, such as warmth and closeness of affectional ties, and level of active emotional support within the family network; and, (c) contextual factors, such as social support, and characteristics of school, work and church settings.

Because protective factors include personality traits and family, community, and environmental characteristics, it is difficult to compile a universal list of factors appropriate to all groups of people in very diverse contexts, especially when the nature or the composition of those categories includes diverse cultural dimensions [16]. For example, self-efficacy is a commonly cited protective factor [13,14,17], but few studies describe the nature of self-efficacy and how it works to protect American Indians or Alaska Natives. Hobfoll, Jackson, Hobfoll, Pierce, and Young [18] expanded our understanding of how efficacy may differ in a collectivist culture. A measure of communal mastery developed for the Hobfoll et al. study, but not a standard self-efficacy measure [19], predicted lower depressive mood and anger among American Indian women in stressful situations. Research among other ethnically diverse populations, including work with indigenous people in Kauai [15], Asian-Americans [20], and culturally-diverse inner city populations [21,22] similarly highlight the importance of cultural factors in the understanding of protective processes.

Triadic Influence theory (TI) [23] provides a multi-level, multi-factorial model for understanding protective factors in sobriety that both integrates constructs from other theories on alcohol use and abuse, and provides a conceptual framework for interventions [24]. However, Petraitis, Flay, and Miller [25] noted that there has been limited research on protective factors within a TI framework associated with race and ethnicity. The limited existing research on the role of cultural factors within protective processes from substance abuse among American Indians and Alaska Natives has focused on cultural identity processes and has yielded mixed findings. Beauvais and Oetting's [26] review of research suggested high levels of cultural identification function as a protective factor from substance abuse among American Indian adolescents, and Schinke et al. [27]found bicultural skills training an effective preventive intervention against substance abuse for this population. However, other studies of cultural identity and substance abuse have found no relation [28], or a positive relationship for women [29]. Oetting, Donnermeryer, Trimble, and Beauvais [30] concluded that simple relationships between cultural identification and substance abuse are unlikely to be found given four potentially overlapping considerations. First, members of an ethnic group vary on level of cultural identification, which may effect conformity to substance use norms. Second, substance abuse may originate from norms socialized in the subculture and differ from those of the larger ethnic group. Third, cultural identification and substance use norms may differ in different contexts. Fourth, cultural identification may originate from primary socialization sources that are different than drug use norms.

Instead of attempting to study cultural factors through measurement of identification with Alaska Native culture, the narrative form of the qualitative study reported in this paper allows for an alternative approach involving the generation of hypotheses on ways in which specific culturally mediated processes are conceptualized as protective by the members of the culture themselves.

In summary, there is a need for research that examines the resilience experience of Alaska Natives who lead sober lives, and in particular, for research that includes an examination of the role of cultural factors in the protective process. In order to provide the rich description necessary to understand the range of experience and cultural processes of Alaska Natives who never drank abusively or who have recovered, qualitative methodologies are used. The goal of this study is to generate a theoretical model [31] of protection grounded in the experience of Alaska Native people that could inform the development of culturally anchored prevention approaches. Aligned with this goal, in this article we focus on Alaska Native pathways to the sobriety outcomes of abstinence and nonproblem alcohol use. Our analysis of the recovery group in this study is therefore restricted to identification of unique attributes within the abstinent and nonproblem drinking group not found among the recovery group. Future research will explore Alaska Native pathways of recovery from alcohol abuse.

## Methods

### Sample

A purposive sampling procedure was used. Selection criteria were established by the PA Coordinating Council, a statewide group consisting of Alaska Native community leaders, individuals involved with grassroots Alaska Native sobriety movement efforts, and Alaska Native substance abuse services providers, who functioned as co-researchers in the participatory methodology. The Council distinguished three groups of interest: (1) lifetime abstainers (LAs) defined as individuals who have never drank more than two drinks per year, (2) non-problem drinkers (NPs) who report drinking alcohol with no problem and score less than 12 on the lifetime total consequences score of the Drinkers Inventory of Consequences for Alaska Natives (DrInC-AN)-a culturally adapted version of the Drinkers Inventory of Consequences (30), and (3) five years or greater of sobriety (5+) who identified themselves as recovered after a serious problem with alcohol, scored greater than 12 on the DrInC-AN lifetime total consequences score, and reported abstinence for at least five years. The project goal for Phase I was to select 36 participants with equal representation from the five Alaska Native tribal groups-Aleut/Alutiq, Athabascan, Inupiaq, Tlingit/Haida/Tsimshian, and Yup'ik/Cup'ik,-balanced by gender, age, and sobriety group status, and to oversample 12 additional interviews from the Yup'ik because Phase II measurement development would focus on this group. PA utilized nomination and snowball procedures to identify potential participants. Age representation was categorized into three age groups: 21 to 30, 30 to 55, and 56 and over. These age ranges were selected by the Council as indicative of culturally significant age ranges, marking indigenous age transitions from young adulthood to middle adulthood to elder. The Council selected these three sobriety categories to maximize our ability to discover potential protective factors as well as recovery factors, together which would define broadly resilience factors used by Alaska Natives in dealing with alcohol. Consultants from the respective tribal communities, the regional non-profit corporations, area health service providers, and other Native political organizations nominated individuals for participation, who then nominated others. Additionally, radio shows, advertisements, and newspaper articles solicited volunteers. This yielded 152 volunteers. Because our Council indicated it would be culturally inappropriate to not interview people following their offer to tell their life story to the project, PA offered interviews to all volunteers, and 101 completed the entire interview process. The results presented here analyze 37 long life history interviews and 14 briefer interviews on sobriety experiences. These participants were distributed across tribal group affiliation (Aleut/Alutiq-6, Athabascan-7, Inupiaq-6, Tlingit/Haida/Tsimshian-6, Yup'ik/Cup'ik-26), and the three sobriety types: LA - 10, NP - 19, and 5+ - 22, with proportional representation of the long life histories by gender and age in each sobriety category. In addition to over-sampling from the Yup'ik cultural group for life history interviews, 14 Yup'ik briefer interviews are included in this analysis in order to maximize the generalizeability of the findings to this cultural group, as the next phases of PA involve the development of measurement instruments and preventative interventions in regions of Alaska that include a Yup'ik majority.

Sixty-two percent of participants spoke English as a first language and 48% their indigenous language. Eighty-two percent had been married at one time, with the average length of marriage being 10 years. At the time of the interviews 57% remained married. Participants' immediate families averaged 3 children. Participant incomes ranged from below $10,000 to over $100,000 per annum with the mean at $46,800. Most participants had graduated from high school (84%) and education ranged from no school to doctoral degrees. Of those who had recovered from alcohol abuse/dependence, mean years of sobriety was 17.5 years.

### Procedures

PA was approved by the Institutional Review Board at the University of Alaska Fairbanks prior to participant enrollment. Nominees were contacted initially by phone, the purpose and structure of the interviews was described, and participation invited. Preference for location of interview, gender of interviewer, indigenous language or English interviewer, and interviewer that they knew or did not know was established. Interviewers were trained in the interview protocol, including protection of human participant procedures, prior to this contact.

Life history interviews followed an open-ended for long life histories (LLH) or semi-structured format for brief life stories (BLS). The mean for LLH was 173.5 minutes (SD = 87.5), median was 159.5, and mode was 141.9. For BLS the mean was 119.5 minutes (SD = 49.5), median was 110, and mode was 106.5. Range for LLH were 20 to 452 minutes and for BLS were 45 to 272 minutes. The interview protocol elicited lifespan information with a focus on what the person considered most important in their process of sobriety. The intent was to garner rich detail about each person's life story. Briefer interviews were semi-structured. Questions addressed specific issues including the role of culture, spirituality, role models, parenting, and the methods of coping that individuals utilized to either not abuse alcohol or to recover. However, it is important to note that Alaska Native narrative patterns [32] at times overrode the distinction between these interview types and participants often responded to both formats similarly in time duration and style of discourse. Many participants tended to respond to either question format with a narrative, and did not distinguish more structured questions from less structured ones, e.g. "When did you first drink and what was your experience like?" in contrast to, "Tell me about your life in as much detail as possible from whatever point that you wish?" would often be answered in the same way and expanded upon equally. Our sense was that older participants in particular would often respond to either type of question by telling their entire life story. Additionally, we noted the length of the interviews also often varied by the experience of the interviewer and/or how the interviewer responded to the content of the interviews. For example, some interviewers felt it was best to close off interviews that began to bring out too much emotional material, whereas others with more clinical experience were more comfortable in moving through emotional material, framing and containing it, and then move on to other material. Interviews were recorded digitally using mini-disk recorders. At interview conclusion, participants completed a demographic questionnaire and the DrInC-AN,

### Analysis

Our analytic approach combined elements of grounded theory analysis [31] with recent methodological advances in team-based coding and analysis [33] and consensual qualitative data analysis [34]. Interviews were verbatim transcribed, reviewed by the interviewer, then, in the case of the life history interviews, the transcript was mailed to and reviewed by the participant for accuracy, additions, or changes. The following describes the analytic process from which a heuristic model of protective factors in Alaska Native sobriety emerged. Although the analytic structure is presented in stages for exposition of its elements, the analysis in practice functioned in an iterative process through multiple passes through stages, involving continual reassessment of inferences and analyses.

#### Step 1: Memoing

Each analysis team member memoed the recordings of assigned interviews while also making additions and corrections to the transcripts for fidelity to the recorded interview. Memoing entailed three steps: (1) open coding identify possible codes, (2) connecting codes through overarching themes, and (3) documenting how codes and themes fit possible theories of protection. Team members then read all memos. Additionally, some of the team members shared their memos with the participant to gather feedback on the accuracy of their perceptions. Changes to the coding and analysis were made to reflect the perceptions of the participant. Most participants made no changes to the transcripts or small changes to the transcripts. A small number made changes by adding material or deciding to delete material, e.g. a number of individuals dropped names of people that were in the interview. A few added material that they had remembered. We gave the participants their verbatim transcripts (with all pauses, false starts, "ahs", etc.) and discovered participants were often embarrassed by their unedited nature. We learned immediately we needed to explain the nature of the transcription process and its intent, and that their interviews would not be published in such a form (participants' interview transcripts were confidential, but several participants expressed a cultural value in their desire to have their interviews made available to others who may be struggling with alcohol themselves and find them helpful). An initial set of codes and overarching themes or domains under which the codes clustered was identified and then systematized in an initial draft coding manual.

#### Step 2: Open coding and coding manual development

Two research team members continued to read and open-code interviews. The team met periodically with Gerald V. Mohatt, Principal Investigator, who also coded a number of transcripts, to discuss coding discrepancies and refine coding rules. The goal at this stage was inclusive not exclusive, and to add as many codes as possible; therefore, we did not limit ideas. We spent much time operationalizing definitions in order to ensure that each code was clearly distinguished from others and could be reliably scored using the codebook criteria. This was done through hours of discussion, with final agreement regarding the definition of each code arrived at between the PI, the research Project Director, and at least one of the Co-Investigators or research assistants. This process resulted in 220 separate codes organized under 25 hierarchical domains. Coding reliability was enhanced in the revised coding manual through development of definitions for each code, along with examples of the code in use and decision rules where appropriate.

#### Step 3: Coding/content analysis and codebook refinement

The research team trained coders to code using AnSWR software [35] and content analyze the remaining transcripts. Inter-coder reliability between coders was assessed on every seventh transcript. What represents adequate inter-coder reliability in qualitative research continues to provoke divergent viewpoints in the literature. Miles and Huberman [36](p. 64) suggest that final inter-coder agreement in qualitative data analysis should approach or exceed 90%, though Stein[37] recently published a study where she used less than 80% agreement. Moreover, simple proportions do not account for the possibility that coders might agree due to chance, which is a function of the frequency or infrequency with which a code appears [38] and therefore provide a biased over-estimate of the true level of agreement. To correct for this, we used the *kappa *statistic [39]. Carey, Morgan, & Oxtoby [40] judged that a *kappa *less than .90 indicated a problem with agreement in the way a code was being used in qualitative research. However, insistence upon very high levels of reliability can also have the effect of diminishing validity [41], and this is a particular concern in discovery-based research such as that of the present study. Therefore, we adopted minimum criteria for the 25 hierarchical categories of *kappa *.90 or greater, and coding of the 220 lower level categories of no less than .60. *Kappas *ranged from .60 to .81 for all lower level categories, and all hierarchical categories were at .90 or above. The team continued to reconcile divergences in coding, refine coding categories, open code, and revise the codebook. Previously coded transcripts were recoded, using the revised codebook.

#### Step 4: Cultural auditing

The team submitted a sample of transcripts to the PA Coordinating Council as part of a cultural auditing procedure. The co-researcher role of this Council, which included members of all five Alaska Native tribal groups interviewed by the project is described elsewhere [5]. The Council collectively open-coded five transcripts from participants selected from all three sobriety groups. Council members coded the transcript of a participant from their own cultural group. The Council convened to discuss their coding and address specific research team questions; such as, have we identified and labelled the codes appropriately. This cultural auditing process moved the team forward in understanding the narratives from a more culturally grounded perspective.

For example, Council members understood "being a role model" within the context of the cultural value of contributing to the good of the family or community, and not merely in terms of individual achievement. The Council also indicated that we should add codes such as shame, praise, and pride to our coding system, and elaborated on their definitions. An overall comparison of the coding and domains generated by the Council with those of the research team displayed high levels of consistency, along with selected important divergences which were discussed to mutual understanding, then adopted by the research coding team.

#### Step 5: Generating theories through a consensual analytic process

Team members next identified how coded segments clustered and interacted, generating potential theories on protective factors through comparison of the life histories of LAs and NPs to 5+ individuals. The team discussed multiple theories, and reconciled potential theories to case histories of non-agreement through revision or abandonment of the theory.

#### Step 6: Developing and refining a theoretical pathway to sobriety

Team discussions were summarized and synthesized by the principal investigator into competing models. The team reread transcripts, discussed and refined models, converging on one model that best fit the majority of transcripts, which was then presented to the PA Coordinating Council. The Council added refinements and culturally grounded elaborations to this model.

#### Step 7: Doubling back

The team re-read transcripts and reassessed the model, refining and elaborating elements until consensus that the full set of transcripts supported the model. As part of this process the team enlisted the Cuiliat Group of Yup'ik speakers, who were our cultural consultants, and would also assist us in the Phase II measurement development. Translating each of the protective factors into Yup'ik forced us to clarify definitions and ensured that they differentiated culturally specific dimensions of each protective factor. For example, from this process the importance of collective group factors became clearer.

### Methods for Verification

In qualitative research, the analogue for validity in quantitative research is often termed *credibility*, which can be defined through (1) the confidence that can be placed in the data and analysis [42,43], (2) how well the conclusions from the data analysis are grounded and supported in the data [44], and (3) the degree to which the descriptions and analyses provide an understanding of the experience studied [45]. In this study, several methods [36] were used to enhance the credibility of the findings: prolonged engagement with the participants resulting in rich, thick description; initial memoing of each narrative prior to coding; confirmation of the narrative and its transcription, and of the memoing, through checks with the study participants; team data coding with ongoing reliability checks and refinement of the coding system; triangulation through the use of multiple data sources and multiple co-researcher perspectives; negative case analysis, or the examination of events and perceptions that did not fit emerging themes; cultural auditing of the coding and interpretative process; and team-based consensual analytic processes. Examples of triangulation included sending transcripts and memoing to the participant,, discussion of the memoing and transcripts with the Council, and the parallel discussions within the research team, which provided three typically converging perspectives on the analysis, along with recognition and discussion of discrepancies whenever they occurred, to the point of mutual understanding, and resolution and agreement. Depending upon the specific theme that was divergent, action could involve reworking of the coding theme to make it more congruent, dropping the theme as an unreliable code,, or addition of a new theme that was not seen by the research coding team, but was identified by others who analyzed the transcripts. Given the multiple cultural perspectives, this provided rich, deep, and inclusive coding categories allowing for the generation of multiple hypotheses regarding themes and the connections between them in the life stories.

### Generalizability

The research aim of the PA study was discovery-based, and not proof through hypothesis testing and falsification. Our objective was to characterize the types of protective factors utilized within this purposive sample, and not to generalize to all Alaska Natives or American Indians. Our goal was to generate a heuristic theory that would suggest testable hypotheses that could later be investigated in a larger, population based study, using measures developed in Phase II. We also hoped to offer ideas to services programs regarding variables that they could test for effectiveness in prevention or treatment.

## Results

**Figure 1:**
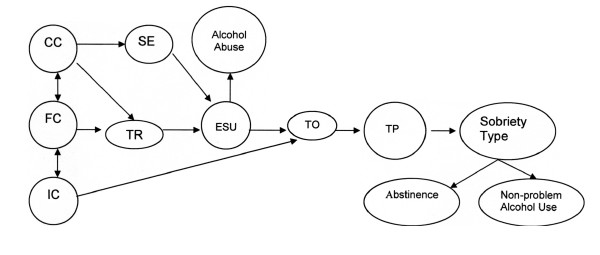
Heuristic Model of Alaska Native Protective Pathways ***Key*. CC (community characteristics) ***Yuut cayarait *includes the way the community organizes family, school, and community activity, and enforces alcohol policy and the drinking status of the community, CC includes role models, opportunities, limits, and safe places. **FE (family environment) ***Ilakelriit cayarait *includes family functioning in such areas as cohesion, conflict, recreation outlets, moral-spiritual focus, and home organization. Factors included parent-child relationship, affection and praise, transmission of expectations, safety and protection from harm and models of sobriety. **IC (individual characteristics) ***Yuum Ayuqucia *are belief in self (communal and self-mastery), wanting to contribute to others and *Ellanqaq *(Yup'ik mindfulness and awareness. **SE (social environment) ***Yuuyaraq *includes role models and social support from extended family, peers, and other adults outside of immediate, nuclear family. **TR (trauma) ***Akngirneq *includes sexual abuse, domestic violence, and death of loved ones. It includes being a victim and observing others being a victim. An individual's perception of trauma is critical, as is the meaning they attach to their experience and how they respond to it. **ESU (experimental substance use) ***Meqerraaryaurtellemni *are early experiences with substances, including alcohol, prior to the establishment of use patterns or abstinence. **TO (thinking it over) ***Umyuangcallemni *involves reflecting on one's experience and developing a personal life narrative. **TP (turning point) ***Ayuqucinellemni *comes out of this reflective process and leads to a decision about how the person will use alcohol.

Using the above process we first identified a set of factors protective from alcohol abuse. We use the direct words of participants to illustrate each to allow the reader to move through the process in a manner similar to the research team. Each protective factor in the model is translated into Yup'ik, the indigenous language of the group we plan to collaborate with on an intervention program. The complete Heuristic Model of Alaska Native Protective Pathways can be found in Figure 1. The mode represents a culture specific mapping of protective processes and as such, is presented in a format that allows for hypothesis testing using quantitative methods. The model is theoretical and heuristic in nature, and shows postulated relationships between factors consistent with Triadic Influence Theory, rather than empirically supported causal factors. We describe below each protective factor, along with its relationship to the model and function.

### Community Characteristics (CC)

*Yuut cayarait*. Participants described the context of the community that protected them during childhood and provided a sense of security. As one participant indicated, "I guess, my life as a child was pretty much sheltered...so, as the expression goes, the village was my oyster then." Protective communities possessed *role models *for the proactive caring of others that exemplified a sense of a collective responsibility for the care of children, or, as another participant described this, "That's also what I remember is people taking care of us even if we're not their children, they looked after us, and they corrected us." Participants described how protective communities provided both opportunities to learn and alternatives to drinking. One young man described how the community school gave *opportunities *to travel, engage in sports, debate, and engage in student leadership that gave him ideas about college and careers. *Opportunities *were also often contextualized in ways the community helped children through important culturally defined transitional rites in the development of adult roles: "They still do this community sponsored moose hunt. They go out and they go hunting for the moose and for a lot of young men that is the time that they have the rite of passage. This is their first moose. And in the beginning when it started out it was just the men, just boys were allowed to go. And it evolved into a community wide project and it does include girls. And the whole community is involved because they'll go and they'll come back in and they'll have a big potluck and it's the rite of passage for he who caught his first moose. Everybody gets to participate. He gets to provide for his community, you know for the first time and that is something that he can do."

One of the most important community protective factors related to how the community established limits. While some individuals discussed the local option laws that allow some communities to vote to regulate or ban alcohol, a larger number discussed how significant individuals in the community took a personal stand to protect children from alcohol-related harm. What was fascinating was in which community characteristics were frequently embedded within the context of the family, and occurred within the interface between the family and the community. A vivid instance of this is described by a middle-aged woman recalling her childhood: "When I first was aware of somebody drinking, I was already nine years old. And I never saw anybody drunk before. ...And my father stood up, and he said no; he just let him turn around and he walked out with him. And then I heard him out there, 'Don't you ever come in my house like that.' We asked my mom, what is wrong with that man? And she would never tell us; she would say in due time you will know. In your own time, you will know." Here we see the individual actions within a family as an important component within a community-wide expectation regarding the setting of limits upon alcoholic behavior, reciprocally mirroring and contributing to a community standard.

Participants reported how they were exposed in childhood to adults that abused alcohol. Protective communities had *safe places *children could go to that prevented them from becoming victims of alcohol-related violence. Most often the safe place was with a close relative, but it could include a friend, teacher, or member of the clergy. As one participant described: "I like the way my grandma took care of me when I was small. Her house was always clean, everything smelled good. It was always a safe place to go to. And I have realized after I got my own place and became an adult, that my home, to other people, was always a safe place to go to."

*Family Characteristics (FC)*, *Ilakelriit Cayarat*. In the words of one participant: "In the Native community families are tied together in a certain way that they're close. And it doesn't matter who you are, we're tied together like a woven hat." This interdependence of family and community highlights both the kinship and collectivist [46] nature of Alaska Native communities. The most fundamental of the protective family factors described by participants was the nature of the caregiver relationship: An *affection and praise *that included important culture specific elements providing children a sense of being valued appears in the following narrative: "And I remember my grandparents bringing us to other elders' homes, just to introduce us to them, because our grandparents were proud of us, and they wanted to share us with the elders in the community. So they brought us to the elders and let us visit with them. I remember when we started hunting and fishing, we got a lot of praise, and even more praise than today, from our relatives and elders. You know, if an elder found out that you caught your first rabbit or your first moose, everybody praised you for that. And it helped to build up the esteem."

Another quality of the caregiver relationship was a sense of being treated as *special*, as very important to the family. One participant noted: "So I grew up to be pretty special, only because I was the only girl of my family. My older brothers took very good care of me. They treated me well." Others who avoided alcohol problems in their lives recalled being told they were to become healers or shamans, or would have similar important roles in the community, and were encouraged to live in a way that prepared them for this role.

Families also provided *safety/protection from harm*. In addition to simply providing a place of safety, caregivers also established limits and enforced them for the good of children. One narrative related the importance of modeling values through the power of both words and action: "I would put the kids to bed and make, you know, put them to bed and make sure those people that were there, some of them I would kick them out and other ones, a lot of times I would let them go, say 'Go drink somewhere else. This is not the place to drink."'

Participants who never developed a drinking problem also described *models of sobriety *in the family who taught them explicitly about how to deal with alcohol: "So my Dad was a non-drinker. And he said when I was eight year old he say, he sat me down, and he told me he said, my son being the oldest in the family, he said, there is something that I want you to do for me. And he said, I want you to carry a torch for me, a torch that you would say that all of my life I wouldn't drink and I wouldn't smoke. He say I took his word for it and he say, I want you to do the same for me. Carry that torch for me. And I guess that's the biggest thing you know that right there and then I thought okay."

Protective families also actively engaged in *transmission *of the expectations they had for their children: "We were a poor family as any village people. But things were happy when we were growing up, and our Mom very seldom went out to work so she was home with us a lot. And my dad would talk to the boys about what's expected of them when they grew up, and how to take their place in the community or in their tribal relatives, how everything worked together. So that's how we all grew up." Many of these protective factors mirror each as interdependent community/family systems that protected children from exposure to alcohol abuse and alcohol related violence.

### Individual Characteristics (IC)

*Yuum Ayuqucia*. Protected individuals displayed a set of characteristics that included a preference towards a cognitive style of thinking through reflectively about what one will or will not do. This reflective style allowed self-control around alcohol use and decisions to immerse oneself in activities that avoid or are incompatible with alcohol use: "But, like I said, it hasn't bothered me – drinking hasn't bothered me. I don't know if it will. In my head – in my mind, it never will. I'm – I'm a positive person and that's the way I like to live my life, is live positively and things go smoother that way. But, living a Yup'ik life, just in general, doing all the traditional activities that we do on a daily – day-to-day basis here in the village, this keeps me away, makes me not think about it."

Participants describe this reflective process as part of a collectivist, other-centered orientation specific to Alaska Native cultures. One participant talked about *wanting to be a role model*: "And I had made a choice when I was ten or eleven to not drink alcohol, to remain sober and to show my brother, my sister that there is something different to do besides drinking and alcohol." The sense of responsibility within a kinship network led to a desire to *give to others – contribute*: "I think he [father] meant that I was going to help people sort out their lives, help them to understand, that you know, be a good listener for them, and counsel them when they need it, or at least let them know they have tools to help themselves.

In order to give and contribute one must have a fundamental sense of one's own capacity, a *belief in self*, as a competent individual. One participant describes: "Like I mentioned, my parents, from as far back as even both of us can remember, I have always been an adult to them. I have always talked to them. Even like when I was ten years old, I talked to them like I was an adult, meaning I listened to them, I didn't talk about silly things. But we were able to converse, and so they treated me like an adult...that gave me the choice to do what I wanted and also to make the decision not to drink."

Some participants described a sense of mastery as knowing and caring for oneself and one's capacity to endure. In the words of one Alaska Native person: "My mother taught me too much to love myself. I've always felt I was a very strong person. I have been able to put up with a lot of shit." However, important differences in mastery emerged between NPs and LAs. NPs often described a sense of efficacy and self-actualization focused more on self-confidence and independence than responsibility to the family and community. One traditional Yup'ik elder NP described how he took the initiative in his socio-cultural education. "Yes I learned on my own. Whenever I am going to construct something I would look at it from all sides and memorize it. When I was about to construct a large boat fashioned after one that is manufactured, I looked at a finished one from all sides and then I constructed it without anyone guiding me. I was not given a lot of advice by anyone." In contrast, for LAs, efficacy was described in more socially embedded terms better labeled as communal mastery [18,47], or a sense that one masters situations best by joining with others.

In this way, several of the life stories describe a socialization process within interconnected collectivist community and family structures that foster becoming aware of how one's actions affect others, described as an *awareness of consequences*: *ellangneq*. *Ellangneq *is a Yup'ik concept, but similar elements appeared throughout many of the narratives across all the Alaska Native cultural groups. The child learns that reciprocity exists between individual actions, and the good of the community and family: control over one's own actions can affect others positively. *Ellangneq *is this culturally valued awareness of the consequences of one's individual actions upon the whole. This special type of awareness is incompatible with intoxication; intoxication only reduces awareness and the ability to control oneself and one's own life, thereby engendering potentially negative reciprocal effects on family, community, and others. In the words of a Yup'ik LA, "But at that time I had already decided for myself that I wasn't going to drink. Part of that had to do with getting out into the woods. And that was part of my reason for refusal. Why would you want to go out and drink and kind of get out of your mind, loose mental control? You know I had so much fun doing the things I wanted to so I wanted to be aware of what I was doing."

### Elaborating the Protective Process

Community and family protective characteristics lowered exposure to alcohol and alcohol-related trauma, or moderated the negative impact of traumatic experiences. They also fostered individual protective characteristics such as sense of mastery, awareness (*Ellangneq*), and a sense of responsibility to family and community.

Nearly half who never drank abusively describe directly experiencing or frequently observing significant trauma during childhood. *Trauma *and/or trauma exposure (TR), *Akngirenq*, included the death of loved ones or other unexpected and intense loss, witnessing domestic violence, or the experience of child abuse including sexual abuse. The pathway of participants who did not use alcohol as a coping response to trauma was facilitated by the protective community, family, and individual characteristics identified in the model, along with the youth's *social environment*, (SE) *Yuuyaraq*, including the presence of healthy, non-alcohol abusing *role models *and *social support *for lifestyles free of alcohol abuse from extended family, peers, and other adults outside of the immediate, nuclear family. Social environment is a subset of community characteristics specific to the time in youth when *experimental substance use *(ESU), *Meqerraaryaurtellemni*, begins, that functions as a support during periods of ESU or in times of crisis such as the experience of trauma. A male who had experienced significant family trauma described this:

"I have a Russian Orthodox priest who's going to wed us in a civil ceremony. And I asked him when I was 15, 'If I ever get married, will you marry me?' He is also somebody who was a mentor for me as a kid.... I think that he was there for me at the right time. Especially, I think, and I probably don't remember a lot of things that happened at that age, but I knew that there was somebody who I could look to."

A period of ESU was quite common in the narratives; a majority of NPs and several LAs engaged in ESU. This typically occurred in early or mid-adolescence, after which the decision to drink responsibly or not drink was made. Consistent with a worldview imbued with concepts allied with that of *Ellangneq*, NPs and in particular many LAs who tried alcohol decided in youth after ESU, or after the experience of significant alcohol-related trauma, that the consequences of alcohol did not fit with how they wanted to affect others. Though even in the presence of multiple family, community, and individual protective factors, children would often still engage in a period of ESU, the outcome among NPs and LAs who experienced these protective factors was a conscious decision, a *turning point *(TP) *Ayuqucinellemn*, that virtually all identified as a pivotal event in their narratives, to either not continue to use alcohol or not use it in a manner that led to abuse. This turning point typically occurred as part of a reflective process of *thinking over *(TO), *Umyuangcallemni*, one's personal experience with alcohol. As one NP described:

"Later on after I graduated from high school I still knew I didn't want to be a drunk or you know, get drunk or look all ugly and do stupid stuff. (...) I didn't want to not know what I was going through. I wanted to be totally aware of my every live moment and I wanted to be in control of everything that I was doing. And so I think that's when my responsible drinking started." Through this process of thinking over and turning point, LAs and NPs composed a personal life narrative in which they were in charge of their lives.

Figure 1 shows community, family, and individual characteristics reciprocally influencing each other. Strong, cohesive communities support the development of healthy families; together these institutions provide the networks of social support that develop a set of individual characteristics that enhance resilience. Strong and positive communities and familial relationships also decrease the likelihood of alcohol-related trauma exposure. They additionally are part of the development of a social environment from which individuals can seek support or resources if trauma is experienced. This occurs in part through development of individual characteristics that enhance the likelihood of a response to trauma or ESU experience that involves thinking over (TO) the experience and the broad and reciprocal consequences of one's actions. This reflective process (TO) facilitates a turning point (TP) in LA and NP outcomes, resulting in a decision to not abuse alcohol, in affirmation of a life goal of contribution to family and community.

## Discussion

We present here a multifactorial and multilevel model for the understanding of the sobriety process of Alaska Natives that lead a life free of alcohol abuse. The model was generated through a participatory action research process, elements of which can be adapted for work with American Indian and other ethnic minority communities. Cultural factors emerged central to an understanding of the sobriety process of Alaska Natives demonstrating the importance of culture as proximal variable [48] in research that seeks to understand sobriety and alcohol abuse with American Indians and Alaska Natives.

The resulting heuristic model for Alaska Native protective pathways is an indigenous explanatory model [49] describing how culturally mediated protective factors interact in complex ways. However, it is also consistent with Triadic Theory of Influence [23] assertions that substance abuse in adolescents is best explained by the interaction of community, family, and individual level variables. The model suggests that community and family build a wider social environment that both supports the youth and interacts with individual factors in the decision to not abuse alcohol following a period of ESU. The mechanism that appears to facilitate the turning point of a sobriety decision is *Ellangneq*, a sense of awareness, mindfulness, and the reciprocity of action developed through the teaching of parents, extended family, and community. *Ellangneq *can be understood as a manifestation of an interdependent [50], constitutive [51], or expanded sense of self [52] found among many Alaska Native and other non-western people that links the individual to a collective, tribal context [46]. Individuals who are socialized within such a context are allocentric [46], with a heightened sensitivity to the effects of their behavior on the whole, and drawing strength from the whole.

*Ellangneq *becomes operative through the actions of family and community. Many researchers have found that a significant relationship with at least one parent is a critical variable in protective outcomes [53], though substitute caregivers can also be of great importance early in the child's life [9,11,54]. This emerged as an important factor in this Alaska Native sample as well; however, the mutual influences of a supportive extended family and community also contributed importantly to resilience. Also important were ways in which security, safety, pride, and affection were experienced through the parent-child interaction, and how the family related to other caregivers to enhance the community network of caregiving.

One important difference distinguished the NP and LA sobriety groups in this sample. For NPs, the resilience process drew from personal stores of self-confidence, self-efficacy, and self-mastery that derived from ability to successfully maneuver within stressful or potentially traumatizing environments [55]. In contrast, for many LAs, efficacy was described in more socially embedded terms of communal mastery [18,47]. One style of mastery is more associated with individualistic orientations, the other with more collectivistic. Future research is needed regarding the generalizability of the group difference in this finding. Nonetheless, the finding highlights important differences between Alaska Native individuals regarding the processes underlying the decision to not abuse alcohol. This finding is of importance both for future research, and in planning interventions for Alaska Native people. The fact that this important difference reflects culturally mediated processes also suggests the decision is itself mediated by variables such as acculturation and cultural identity.

Indeed, cultural factors surfaced repeatedly as important components in an understanding of how social influences within a community and family context functioned as salient protective factors in sobriety for Alaska Natives. As Triandis [46] remarked, "Culture is to society what memory is to the person" (p. 511). In our Alaska Native participants' narratives, cultural processes emerged as much more than immersion in activities, social grouping, or self-perception, imbuing structure and meaning to all aspects of their thoughts and behavior. Even basic components of cultural processes, such as a person's identification with their Alaska Native culture, emerged as complex, situational, and multidimensional, echoing previous critiques of cultural identity research with American Indians and Alaska Natives [56].

## Conclusions

This study presents a heuristic model of Alaska Native pathways to sobriety. What is significant about the model is that it emerged from in-depth study of the experience of Alaska Natives, rather than that of other groups. The model moves current research in the direction of developing a culturally and contextually based explanatory model [49] or emic model [57] of Alaska Native sobriety, because it comes out of the life histories of Alaska Natives and a collaborative analysis process that included Native and non-Native researchers, the community of concern, and the participants themselves, as co-researchers [5]. Tests of hypotheses and path analytic models generated by the heuristic model, and design and investigation of the efficacy of prevention programs based upon the model are important future steps for research. In addition, the findings of this study offer perspectives on the resilience and the sobriety process of indigenous people and more precisely contextualize elements of the Triadic Theory of Influence within one indigenous group.

This initial analysis of the PA data set provides as many questions as answers for our understanding of the sobriety process of Alaska Natives. We hope that as the answers become more clearly defined, those pathways to recovery and resilience walked by the research participants become more known to those in need. The seeds of resilience form a sense of the family and community, a desire to make a difference as one Alutiq elder acknowledges:

"We're not here tomorrow. Got to leave a few tracks around, right? I want to. So my grandkids could say, well, I remember when grandma used to – you know. You know you feel like, hey, you want to be able to leave some kind of memory."

## Competing interests

The author(s) declare that they have no competing interests.

## Authors' contributions

GVM is PI for the research. He led the analysis and interpretation, and completed many of the interviews. SMR completed much of the text analysis, wrote the first draft, and edited many drafts. LT interviewed most of the Tlingit participants, participated in all analysis and interpretation, and edited the paper. JA was a collaborating investigator involved in all aspects of data gathering, analysis, and interpretation. He edited each draft and significantly contributed to the final draft. KH was also a collaborating investigator and edited a number of the drafts. The People Awakening Team completed many of the interviews and assisted with the analysis and interpretation of the data. CH was research coordinator for PA and assisted in model development, analysis, interpretation of data, and editing.
